# Computational capability of ecological dynamics

**DOI:** 10.1098/rsos.221614

**Published:** 2023-04-19

**Authors:** Masayuki Ushio, Kazufumi Watanabe, Yasuhiro Fukuda, Yuji Tokudome, Kohei Nakajima

**Affiliations:** ^1^ Hakubi Center, Kyoto University, Yoshida-Honmachi, Sakyo-ku, Kyoto 606-8501, Japan; ^2^ Center for Ecological Research, Kyoto University, 2-509-3 Hirano, Otsu, Shiga 520-2113, Japan; ^3^ Department of Ocean Science, The Hong Kong University of Science and Technology, Clear Water Bay, Kowloon, Hong Kong, People's Republic of China; ^4^ B.Creation Inc., 5-2 Narihiracho, Ashiya, Hyogo 659-0068, Japan; ^5^ Graduate School of Agricultural Science, Tohoku University, Yomogida Naruko-onsen, Osaki, Miyagi 989-6711, Japan; ^6^ Graduate School of Information Science and Technology, The University of Tokyo, 7-3-1 Hongo, Bunkyo-ku, Tokyo 113-8656, Japan

**Keywords:** computational capability, ecological dynamics, ecological networks, machine learning, neural network, reservoir computing

## Abstract

Ecological dynamics is driven by complex ecological networks. Computational capabilities of artificial networks have been exploited for machine learning purposes, yet whether an ecological network possesses a computational capability and whether/how we can use it remain unclear. Here, we developed two new computational/empirical frameworks based on reservoir computing and show that ecological dynamics can be used as a computational resource. *In silico* ecological reservoir computing (ERC) reconstructs ecological dynamics from empirical time series and uses simulated system responses for information processing, which can predict near future of chaotic dynamics and emulate nonlinear dynamics. The real-time ERC uses real population dynamics of a unicellular organism, *Tetrahymena thermophila*. The temperature of the medium is an input signal and population dynamics is used as a computational resource. Intriguingly, the real-time ecological reservoir has necessary conditions for computing (e.g. synchronized dynamics in response to the same input sequences) and can make near-future predictions of empirical time series, showing the first empirical evidence that population-level phenomenon is capable of real-time computations. Our finding that ecological dynamics possess computational capability poses new research questions for computational science and ecology: how can we efficiently use it and how is it actually used, evolved and maintained in an ecosystem?

## 1. Introduction

Ecological dynamics is driven by complex interactions such as interspecific and biotic–abiotic interactions. Empirical and theoretical studies have shown that prey–predator, mutualistic, competitive and biotic–abiotic interactions are prevalent, and that they play a pivotal role in ecological community dynamics [1–4]. In nature, the interactions shape an ecological network. Information of a node, for example, species abundance or the state of an abiotic variable, can be processed through interactions and transferred to another node in a complex way that is often difficult to be accurately represented by equations. Population or community dynamics includes temporal fluctuations in species abundance and is a consequence of the ‘information processing’. Ecologists have tried to discern rules that govern the ecological dynamics or the information processing.

The terminologies ‘network’ and ‘information processing’ also appear in computational science, but they have been studied from a different viewpoint. In computational science, information processing capability of artificial networks is exploited as a computational resource. Artificial neural networks are represented by a network of neuron-like processing units (nodes) interconnected via synapse-like weighted links (interactions), which are typically classified into feedforward neural networks [5] and recurrent neural networks (RNNs) [6]. A machine learning approach called reservoir computing (RC) is a special type of RNN that is suitable for temporal information processing such as time series analysis [7,8]. In RC, input data are nonlinearly transformed into patterns in a high-dimensional space by an RNN called a ‘reservoir’. Then, a pattern analysis from the transformed patterns is performed in the readout. The main characteristic of RC is that the input weights (***W***_in_) and the weights of the recurrent connections within the reservoir (***W***) are not trained, whereas only the readout weights (***W***_out_) are trained with a simple learning algorithm such as a linear regression. This simple and fast training process makes it possible to drastically reduce the computational cost of learning compared with standard RNNs, which is the major advantage of RC [7,8].

Recently, RC implementation using a physical material has been gaining growing attention in machine learning and engineering fields (physical RC) [9]. A nonlinear, complex information processing capability is embedded in a physical material (i.e. embodiment) [10], and thus one can replace a reservoir in RC with a physical material. For example, a soft robotic, tentacle-like arm can process an input signal from a motor that initiates a movement of the robotic arm, and then the signal transmits through the arm in a way that depends on physical characteristics of the robotic arm such as length, material and shape. Nakajima *et al.* [11,12] have shown that such a soft robotic arm has a short-term memory and can be used to solve several computational tasks in real time. In addition, a recent study has shown that even biological entities such as plants may be used as a physical reservoir [13].

**Figure 1.**
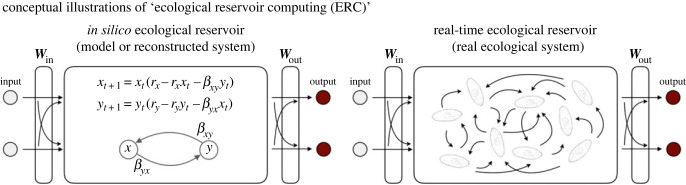
Conceptual illustrations of ecological reservoir computing (ERC) and how in silico ERC is implemented. (*a*) Conceptual illustrations of *in silico* ERC and real-time ERC. *In silico* ERC uses either equations or empirical dynamics reconstructed by empirical time series as a reservoir. In the present study, we mainly investigated the properties and performance of *in silico* ERC using reconstructed dynamics. Real-time ERC uses an empirical ecological interaction network as a reservoir. A node in an ecological reservoir may represent an individual, species or abiotic variable in this study.

Several successful examples of physical RC [8,9,14] imply that we may be able to use information processing capability of other types of networks as a computational resource. Here, we show that ecological dynamics can be used as a computational resource. We call this approach ‘ecological reservoir computing (ERC)’ and implement two types of ERC in this study (figure 1). The first type of ERC is *in silico* ERC; it reconstructs ecological dynamics from empirical time series using a time-delay embedding [15] (i.e. an equation-free ecosystem model) and simulates the system dynamics in response to hypothetical input signals. *In silico* ERC uses the reconstructed dynamics and the simulated responses as a reservoir and reservoir states, respectively, which successfully predicts the near future of chaotic dynamics and emulates nonlinear dynamics. The second type of ERC is real-time ERC; we set up an experimental system that enables continuous monitoring of population dynamics of a unicellular eukaryotic organism, *Tetrahymena thermophila*, in a small chamber. We manipulated the temperature of the medium in the chamber as input signal, and monitored changes in population abundance as reservoir states (i.e. a model-free empirical system). Surprisingly, the real-time ERC has the necessary conditions for RC and is able to make near-future predictions of model and empirical time series. The computational performance of the real-time ERC is currently lower than that of other types of RC, but the finding that untrained ecological dynamics may possess necessary conditions for RC would open up new research directions in computational science and ecology.

## 2. Results and discussion

### 2.1. Reconstructed ecological dynamics as a reservoir

A seminal RC framework, echo state network (ESN), was proposed by Jaeger [7,16]. This model uses an RNN-based reservoir consisting of discrete-time artificial neurons. When the feedback from the output to the reservoir is absent, the time evolution of the neuronal states in the reservoir is described as

2.1 $$\begin{array}{c} X_{t+1}=f(W_{\mathrm{in}}u_{t}+WX_{t}), \end{array}$$

where *t* denotes discrete time, ***X****_t_* is the state vector of the reservoir units, ***u****_t_* is the input vector, ***W***_in_ is the weight matrix for the input-reservoir connections and ***W*** is the weight matrix for the recurrent connections in the reservoir. The function *f* represents an element-wise activation function of the reservoir units, which is typically a sigmoid-type activation function. In the context of a population or community dynamics model in ecology, the weight matrix, ***W***, represents a rule such as prey–predator interactions that govern the dynamics. Therefore, if equations governing the ecological dynamics are known, we can use the system for RC. This is clearly demonstrated in Methods and electronic supplementary material, figure S1 using a simple two-species model system, i.e. Lotka–Volterra equations, in the subsection ‘*Demonstration of the concept of ecological reservoir computing*’ in Methods (the computational performance of the system is low due to a small reservoir size).

**Figure 2.**
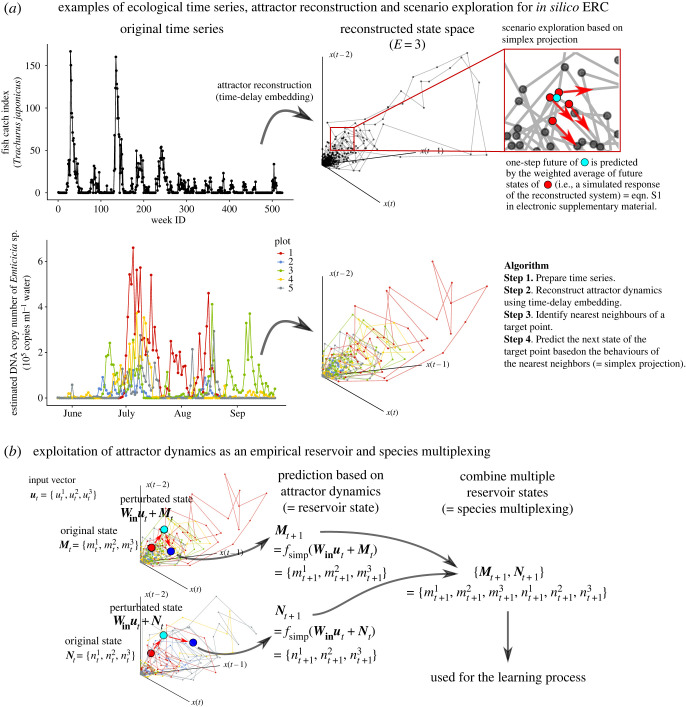
Schematic illustrations of *in silico* ecological reservoir computing (ERC). (*a*) Examples of ecological time series, state space reconstruction and scenario exploration for *in silico* ERC. Two empirical time series are shown as examples: fish-catch time series of Japanese jack mackerel (*Trachurus japonicus*) and DNA copy number time series of *Emticicia* sp. in water samples collected from experimental rice plots (Ushio [23]). Empirical attractor dynamics can be reconstructed by time-delay embedding (embedding dimension = 3). The red inset indicates that the behaviour of a target point (light blue) is predicted by the behaviours (red arrows) of nearest neighbours (red points) (see electronic supplementary material, appendix I and methods). (*b*) Schematic illustrations of species multiplexing using scenario exploration. Simulated ecosystem responses are collected to generate a ‘multiplexed reservoir state’ ({*M_t_*_+1_, *N_t_*_+1_}), which is used for the learning process. Red point in the reconstructed state space indicates the original vector (*M_t_* or *N_t_*), light blue point indicates perturbated vector (e.g. *W*_in_*u_t_* + *M_t_*), and blue point indicates the predicted response of the system by simplex projection (denoted by, e.g. *f*_simp_(*W*_in_*u_t_* + *M_t_*)). As in the main text, the predicted response may be calculated by *W*_in_*u_t_* + *f*_simp_(*M*_*t*_).

Unfortunately, however, we usually do not know equations that govern real, complex ecological dynamics. In other words, although previous studies demonstrated universal rules on ‘model’ ecological communities with explicit equations [17,18], whether and how these formulations accurately represent real ecological dynamics are usually unknown. In this circumstance, an ‘equation-free modelling’ approach enables extracting a potential mechanism that drives the dynamics. Among such approaches, a nonlinear time series analysis called ‘empirical dynamic modelling’ [19–21] may provide a promising way to use an empirical ecological time series as a reservoir. According to a delay embedding theorem [15,22], multivariate system dynamics may be reconstructed from a single time series using a time-delay embedding (i.e. reconstruction of system dynamics by plotting time-lagged coordinates in a multi-dimensional space; figure 2*a* and electronic supplementary material, appendix I) even when equations governing system dynamics are unknown, which is known as state space reconstruction (SSR). One may add one or more variables (ordinates) in the reconstructed state space, allowing simulations of ecosystem response to external forces, which is a forecasting method of empirical dynamic modelling known as ‘scenario exploration’ [21]. Scenario exploration predicts ecosystem response to hypothetical changes in external forces by averaging near-future behaviours of nearest neighbours of a target state in the reconstructed state space [21,24]. Deyle *et al*. [21] developed scenario exploration and predicted how changes in sea surface temperature influence population abundance of Pacific sardine. In the context of RC, changes in sea surface temperature and predicted population abundance of Pacific sardine may be regarded as ‘input’ and ‘reservoir state’, respectively.

We demonstrate *in silico* ERC, as a scenario-exploration-based approach, using empirical ecological time series: (i) fish-catch time series collected from pelagic regions in Japan and (ii) DNA-based quantitative prokaryote time series taken from experimental rice plots (figure 2*a*) [23]. State spaces of the system were first reconstructed using an optimal embedding dimension (*E*) determined following a previous study [25]. Then, reservoir states were calculated as follows:

2.2 $$\begin{array}{c} X_{t+1}=f(W_{\mathrm{in}}u_{t}+f_{\mathrm{simp}}(X_{t})), \end{array}$$

where ***f***_simp_ indicates a ‘simplex projection’ [25], a nonlinear forecasting method that predicts a behaviour of a target state based on behaviours of nearest neighbours in the reconstructed state space. Briefly, if a target state is a state at time t* (= ***X***_t*_), then ***f***_simp_ looks for nearest neighbours around the target state ***X***_t*_, and predicts the future state ***X***_t*__+1_ by calculating the weighted average of the future states of the nearest neighbours (‘*1. Overview of* in silico *ERC*’ in electronic supplementary material, methods). The behaviour of ***X****_t_* is predicted by ***f***_simp_ so that ***X****_t_* follows the rule of the empirical ecological dynamics. In other words, ***f***_simp_ reflects the rules or behaviours of nearest neighbours, and thus may implicitly include the consequences of interspecific interactions such as prey–predator interactions and competitions. Then, a hypothetical input, ***u****_t_*, is added to the state after transformation by ***W***_in_. We choose an identity function as ***f*** so that equation (2.2) can be interpreted as the population dynamics. Alternatively, one may apply ***f***_simp_ after adding a hypothetical input, ***u****_t_*, to ***X****_t_* (figure 2 and supplementary material, methods). In addition, one may easily multiplex reservoir states generated by different species (species multiplexing; figure 2*b*), which improves the performance of *in silico* ERC.

**Figure 3.**
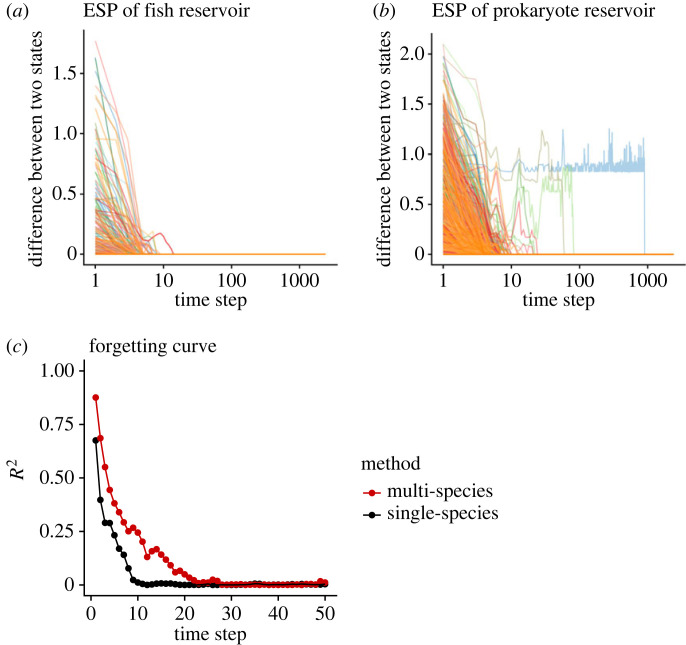
Echo state property and memory capacity of *in silico* ecological reservoir computing (ERC). (*a*) Echo state property (ESP) of the reconstructed fish reservoirs (47 fish species included). For each run (i.e., each species' reservoir), the computation of *in silico* ERC started from two different initial conditions, and the dependence of the state difference on the time step was measured by the Euclidean distance between the two states. Different line colours indicate ecological reservoirs reconstructed by different fish species. (*b*) ESP of the reconstructed prokaryote reservoirs (500 prokaryote species included). *y*-axis in *a* and *b* indicates the difference between reservoir states started from different initial conditions and the difference converges to zero when the same input sequence is used. (*c*) An example of forgetting curves of reconstructed prokaryote reservoir. Forgotten curves of single-species prokaryotic reservoir (*Bdellovibrio* sp.; the black points and lines) and species-multiplexed prokaryotic reservoir (the red points and lines) are shown.

This type of *in silico* ecological reservoir is an equation-free ecosystem model and it possesses a specific memory capacity and shows echo state property (ESP), which are necessary for successful RC (figure 3 and electronic supplementary, methods). Even if initial conditions of an ecological reservoir are different, the difference in the reservoir states converges to zero when the same input sequence is used (figure 3*a*,*b*; uniform random values are used as the input sequence), suggesting that the *in silico* ecological reservoir possesses ESP. In terms of memory capacity, the *in silico* ecological reservoir remembers the information of input data about 10–20 time steps ago (figure 3*c*; species-multiplexed reservoir shows a higher memory capacity). We also measured information processing capacities, which can evaluate the expressiveness of the reservoir in terms of memory capacity and nonlinear processing of inputs, using both species systematically (electronic supplementary material, figure S2 and methods). Interestingly, the analysis on information processing capacity suggests that the *in silico* ecological reservoir has relatively higher nonlinear processing capacity than the linear one compared with the profile of the conventional ESNs (electronic supplementary material, figure S2c). These results together suggest that *in silico* ERC satisfies necessary conditions for RC.

**Figure 4.**
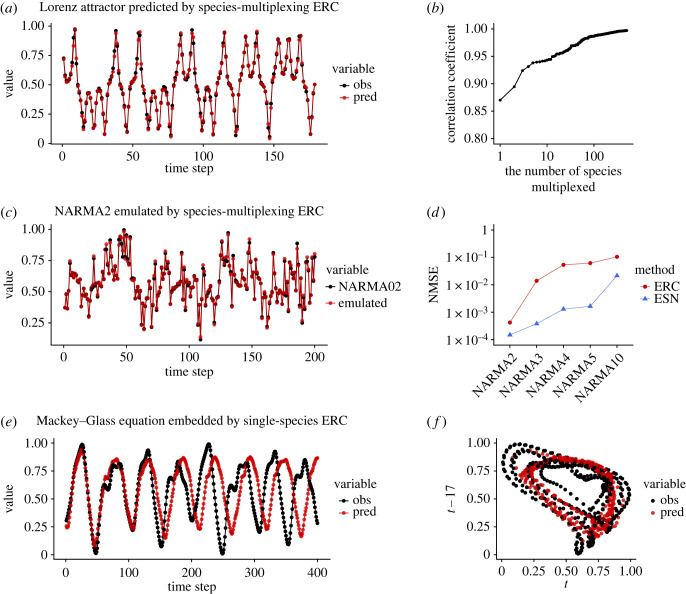
The performance of species-multiplexed *in silico* ecological reservoir computing (ERC). (*a*) Time series of Lorenz attractor (black points and lines) and one-time-step future predictions by species-multiplexed *in silico* ERC (red points and lines). (*b*) Correlation coefficients of observed and predicted values of Lorenz system and the number of species-multiplexed. (*c*) Nonlinear autoregression moving average (NARMA) time series (black points and lines) and emulation by species-multiplexed *in silico* ERC (red points and lines). NARMA is NARMA2. (*d*) Normalized mean square error (NMSE) of the NARMA emulations by species-multiplexed *in silico* ERC (red points and lines) and echo state network (ESN; blue points and lines). (*e*) The closed-loop embedding of the Mackey–Glass equations by species-multiplexed *in silico* ERC. The original attractor (black points and lines) was learned by the *in silico* ERC and autonomous dynamics was generated from time point zero on the *x*-axis (red points and lines). (*f*) Two-dimensional representation of the original Mackey–Glass attractor (black points) and that generated by the *in silico* ERC (red points).

We tested the performance of *in silico* ERC by several standard tasks: prediction of chaotic dynamics, emulation of nonlinear autoregression moving average (NARMA) time series and generation of an autonomous system (Mackey–Glass equation) (for detailed parameters, see electronic supplementary material, tables S1 and S2 and methods). First, *in silico* ERC with species multiplexing accurately predicts Lorenz attractor (figure 4*a*; time series of 500 prokaryotic species in the same experimental system were multiplexed [23]; total reservoir size = 3271; electronic supplementary material, table S1), which outperforms predictions made by the simple two-species model reservoir (electronic supplementary material, figure S1e). Interestingly, the prediction accuracy measured by a correlation coefficient increases with the number of species-multiplexed (figure 4*b*), suggesting that species diversity of a community might be related to the computational capability of an ecological community. Second, NARMA2 (eqn. 8 in electronic supplementary material, methods) can be accurately emulated with species-multiplexed *in silico* ERC (figure 4*c*) though the performance is still lower than that of ESN, a typical RC method (figure 4*d* for NARMA2, 3, 4, 5 and 10; total reservoir size of ESN = 2000; electronic supplementary material, table S1). Third, the Mackey–Glass equation cannot be embedded in a closed loop with *in silico* ERC in our current numerical experiments, but *in silico* ERC generates different attractor dynamics (figure 4*e*,*f*). Altogether, though the performance is still lower than that of ESN, these results show that the method based on scenario exploration can be used as RC and solves several standard tasks. More importantly, *in silico* ERC implies that real ecological dynamics may also be used as a computational resource.

### 2.2. Real-time ecological reservoir computing

**Figure 5.**
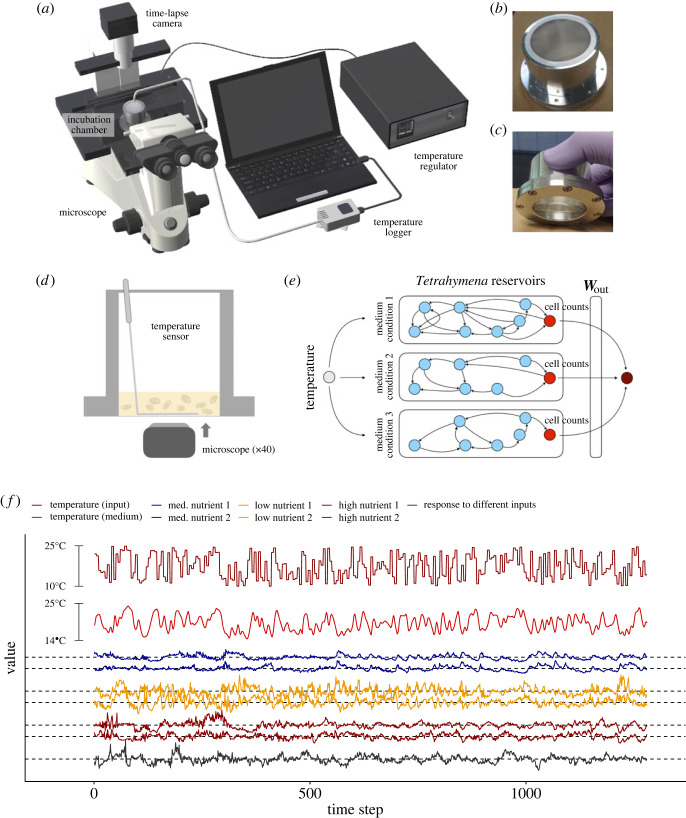
The experimental set-up of *Tetrahymena thermophila* reservoir. (*a*) Illustrations of experimental set-up. (*b*,*c*) Pre-incubated *Tetrahymena* population is maintained in an aluminium chamber. The total medium volume is 5 ml, and the concentration of nutrient is adjusted to change hyperparameters of the population dynamics. (*d*) Cell images were taken from the bottom of the chamber. The number of cells was counted using a custom image analysis pipeline. (*e*) We used 1.6%, 4% and 10% modified Neff medium in the experiments. Temperature information first transmits from the regulator to the aluminium chamber, and then propagates to several nodes in the medium and *Tetrahymena*. The temperature information is processed through complex interactions among temperature, medium and behaviour, and physiology and *Tetrahymena*. The number of cells at the bottom of the chamber may contain the processed information and we use it as a reservoir state. Reservoir states of three different nutrient concentrations were used to improve the performance of real-time ERC (i.e. space-multiplexing). (*f*) An example of random temperature inputs and reservoir states. Time series indicate input temperature (dense red), medium temperature (red) and a population density index (relative residual of the population density) in a 4% (blue line; low nutrient), 1.6% (orange line; med. nutrient) or 10% (brown line; high nutrient) modified Neff medium. Black line indicates population density index of *Tetrahymena* in a 4% modified Neff medium in response to a different temperature input sequence.

In this section, we show that ERC is possible even with real ecological dynamics. For real-time ERC, we set up an experimental system to use the population dynamics of a eukaryotic unicellular organism, *Tetrahymena thermophila* [26] (hereafter, *Tetrahymena*), as a reservoir (figure 5*a–d*; Methods; electronic supplementary material, figures S3 and S4). In the experiment, the *Tetrahymena* population dynamics in an aluminium chamber is monitored by time-lapse imaging combined with a standard particle analysis (Methods and electronic supplementary material, figure S5a). Medium temperature is accurately controlled with a custom temperature regulator at 5 min intervals, which is an input signal of real-time ERC. Although there is only a single species in the system, many factors, including temperature, medium concentration, cell-to-cell interactions and individual behaviours interact to generate complex, nonlinear dynamics [27–29] (figure 5*d*,*e*), which we expected would be suitable for real-time ERC. Thus, the number of cells captured in an image is an outcome of the complex system response to an external force (see Methods for the formulation of the system response in the context of RC), and this system is a model-free empirical system for RC.

To obtain reservoir states of the system and improve its computational capability, we adopted several strategies: data preprocessing, time-multiplexing and space-multiplexing. First, the *Tetrahymena* population dynamics was preprocessed to be stationary and unbiased (electronic supplementary material, figure S5b–d), which represents responses of the *Tetrahymena* population to the inputs and was used as reservoir states. Second, five reservoir states were multiplexed for one input (time-multiplexing; i.e. a reservoir state was taken every minute and an input signal was manipulated every 5 min; electronic supplementary material, methods and appendices II and III). Third, reservoir states taken using three medium concentrations (1.6%, 4% and 10% modified Neff medium) were multiplexed to further increase the reservoir size (space-multiplexing; electronic supplementary material, methods and appendices II and III).

**Figure 6.**
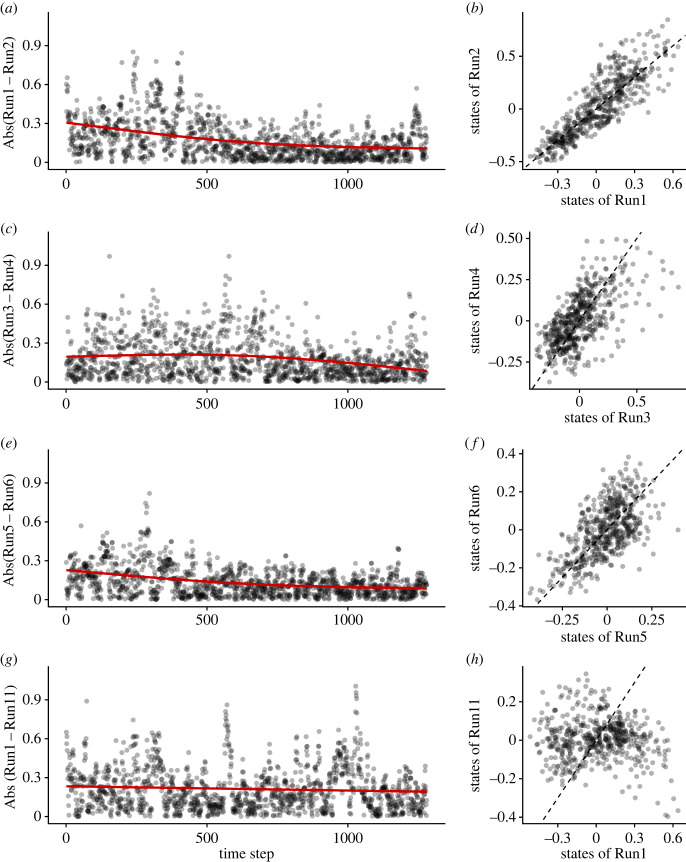
Echo state property of real-time ecological reservoir computing. Two runs for each medium concentration were tested and the input sequence was identical for (*a–f*). (*a*,*b*) Comparison of two reservoir states (i.e. relative GAM residuals explained in electronic supplementary material, figure S5; Run1 and Run2) where the same input sequence was added in 4% Neff medium. Time series plot (*a*) and scattered plot for the last 501 time points (*b*). (*c*,*d*) Comparison of two reservoir states in 1.6% Neff medium (Run3 and Run4) and (*e*,*f*) in 10% Neff medium (Run5 and Run6). (*g*,*h*) Comparison of two reservoir states in 4% Neff medium, but the input sequence was different for Run1 and Run11. Reservoir outputs converged for the identical inputs when the medium concentration was the same (*a–f*). On the other hand, the reservoir outputs did not converge when the input sequences were different (*g*,*h*). Red and dashed lines indicate GAM regression and 1 : 1 line, respectively.

**Figure 7.**
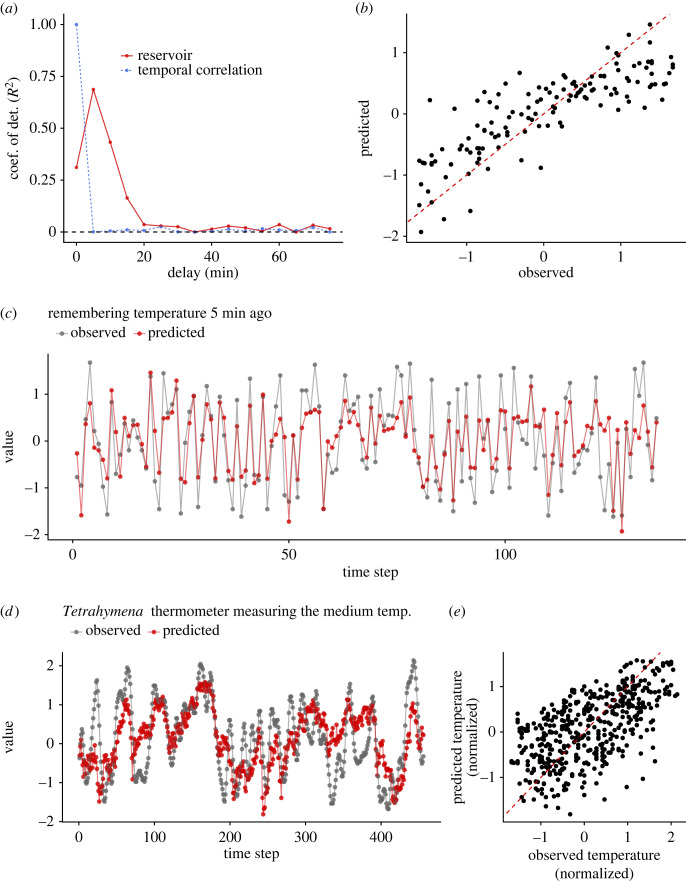
Memory capacity of the *Tetrahymena* reservoirs. (*a*) Memory capacity of the *Tetrahymena* reservoir measured using three time series of population density index (from three medium concentrations) as a training set and the other three time series as a test set (total six time series were used). Red points and lines indicate how well the *Tetrahymena* dynamics remembers the uniform random inputs. Blue points and dotted lines indicate temporal autocorrelations. (*b*) Correlations between observed and predicted values of uniform random inputs (i.e., temperatures set in the regulator). Values are standardized. Red dashed line indicates 1 : 1 line. (*c*) Observed (grey points and lines) and predicted (red points and lines) time series of uniform random temperature inputs. In (*b*,*c*), temperature inputs 5 min ago (set in the regulator) were predicted by the states of the *Tetrahymena* reservoir. (*d*) Measurements of the medium temperatures 5 min ago by the *Tetrahymena* reservoir, suggesting that the *Tetrahymena* reservoir may work as a thermometer (*Tetrahymena* thermometer). (*e*) Correlations between observed and predicted temperature by the *Tetrahymena* thermometer.

We first tested whether the *Tetrahymena* reservoir has a memory capacity and ESP by inputting uniform random values as medium temperature using the three strategies. Examples of the monitoring results with the same input sequence and the same medium concentration for different trials are shown in figure 5*f* and electronic supplementary material, movie S1 (https://www.youtube.com/watch?v=z_QeEka4W3w), which shows a clear common-signal-induced synchronization that is a signature of ESP [30,31]. A different medium concentration generated different population dynamics (figure 5*f*), suggesting that the population dynamics under a different medium concentration may be used as a reservoir with different hyperparameters. We further tested the correspondences between the two runs for each medium concentration, and found that the state differences become smaller when the same input sequence is inputted to the system (figure 6*a–f*). On the other hand, with a different input sequence in the 4% medium system, the population dynamics show a different pattern and the state difference does not converge (figure 6*g*,*h*). These results suggest that the system has ESP [30,31]. In addition, the population dynamics have a specific memory capacity; the dynamics recover the input values at 5–15 min ago (= 1–3 steps ago) (figure 7*a–c*). These characteristics enable the *Tetrahymena* reservoir to measure the medium temperature (figure 7*d*,*e*), showing that, by using the short-term memory of the community dynamics (not the medium), the *Tetrahymena* population dynamics can work as a ‘thermometer’ of the system. Together, these results suggest that the ecological reservoir may be used as a computational resource.

**Figure 8.**
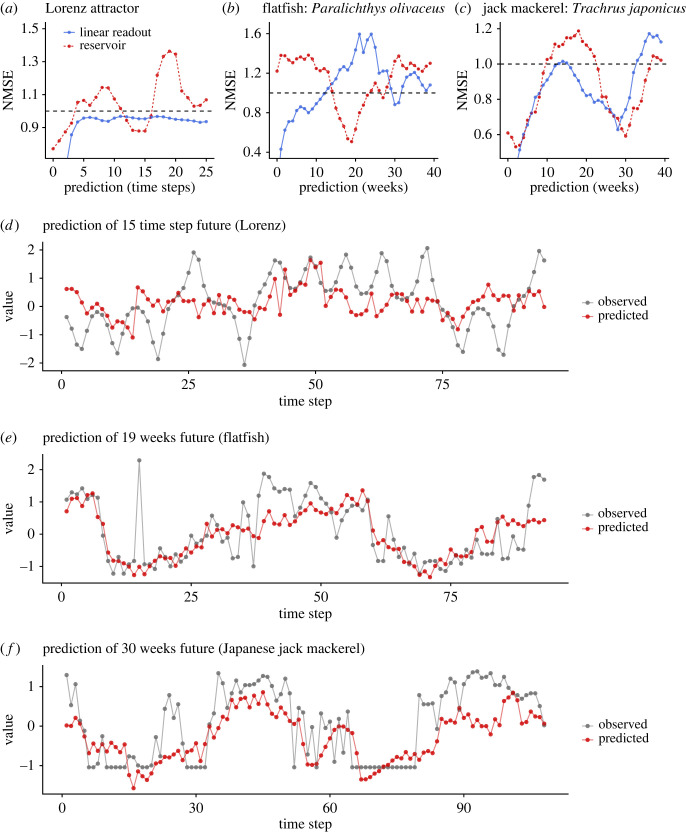
The relationship between prediction accuracy (normalized mean square error; NMSE) and prediction time step. (*a*) Lorenz attractor, (*b*) flatfish time series and (*c*) Japanese jack mackerel time series. Red points and lines indicate predictions by the *Tetrahymena* reservoir, and blue points and lines indicate predictions by ridge regressions. Time series of observed (grey points and lines) and predicted (red points and values) of (*d*) Lorenz attractor with 15 time-step future prediction, (*e*) flatfish time series with 19 weeks future prediction and (*f*) Japanese jack mackerel time series with 30 weeks future prediction.

To explicitly show that the *Tetrahymena* reservoir can solve computational tasks, we predicted three time series: Lorenz attractor (model time series) and two fish-catch time series (empirical time series). As with the uniform random inputs, the same inputs generate similar population dynamics under the same medium concentration, showing ESP of the system (electronic supplementary material, figure S6). By time- and space-multiplexing those reservoir states, the *Tetrahymena* reservoir reasonably predicts the near future of the three time series (figure 8*a–c*; see electronic supplementary material, movie S2 for how the *Tetrahymena* reservoir predicts the near future in real time; https://www.youtube.com/watch?v=SUmkYAnfjFk). The predictions made by the *Tetrahymena* reservoir are more accurate than those made by linear readout at certain time points, suggesting that the computational capability of *Tetrahymena* population dynamics has been successfully extracted and used by the experimental system. The *Tetrahymena* reservoir predicts 15 time-step future of Lorenz attractor, 19 time-step future of flatfish time series and 30 time-step future of Japanese jack mackerel time series (figure 8*d–f*).

### 2.3. Significance, current limitations and future perspectives

Recently, machines built completely from biological tissues, called living machines or biological robots, are gaining attention due to the extreme adaptability and flexibility provided by the protean nature of their donor organisms [32]. Our work shares the same line of motivation and serves as one of the examples of living machines or biological robots. However, ecological dynamics are often more complex and less understood than individual-level phenomena that have previously inspired living machines or biological robots. Perhaps partly due to this complexity, the computational capability of ecological dynamics has not been explored so far. This study illustrates for the first time that ecological dynamics has computational capability that could be harnessed. In this section, we will discuss the significance of the finding, its current limitations and future perspectives.

In the present study, we show the first empirical evidence that ecological dynamics can be used as a computational resource in two ways: *in silico* ERC and real-time ERC. The former provides a numerical framework to quantify the ‘potential’ computational capability of the ecological dynamics and to use the reconstructed dynamics as a computational resource. In the context of computational science, while *in silico* ERC currently has poorer computational performance than traditional RC (figure 4), the potential of reconstructed dynamics as a reservoir is worth exploring as it allows for the transformation of input signals into a higher dimensional feature space in an unconventional manner. In the context of ecology, quantifying the computational capabilities of ecological time series is important for two reasons. First, it provides an efficient way to search for ecological dynamics with potentially high information processing capability in the real world. Second, comparing the computational capabilities with ecological factors (such as species identity, phylogeny and environmental factors) may provide insights into how and why the computational capability of ecological dynamics evolved. For example, communities in a rapidly changing environment might possess higher information processing capacity than those in a stable environment.

The latter, i.e. the fact that untrained ecological dynamics possess necessary conditions for RC and can solve several machine learning tasks, is more intriguing, and its significance is multi-fold. In the context of computational science, real-time ERC is a novel computational framework. Though the computational performance of ERC is still lower than that of the typical RC, other ecological dynamics (e.g. high-diversity community dynamics) with different experimental settings (e.g. different input signals such as light) will possess different reservoir properties (e.g. with/without ESP and different memory capacity) and such ecological reservoirs might outperform the typical RC. To achieve this, the development of methods for efficient (eco)system monitoring is key. In the context of ecology, the real-time ERC enables quantifications of the computational capability of empirical ecological populations or communities. The computational capability may be regarded as an ‘extended’ functional trait of organisms, which should evolve by interacting with biotic and abiotic factors in a natural habitat. As we stated in the previous paragraph, comparing the computational capabilities with ecological factors may provide insights into how and why the computational capability evolved. In addition, identifying responsible genes for the computational capability and designing organisms with a high computation capability would be a fascinating direction. Also, a community with high diversity may potentially have a high reservoir size, which could beget a high computational capability (as shown in figure 3*b*). The high computational capability, high community diversity and stable ecosystem functions might be interdependent, and the potential positive relationship between community diversity and computational capability may add a new value to biodiversity. Lastly, if the ‘closed-loop’ approach as shown in the Mackey–Glass equation in *in silico* ERC (figure 3*e*,*f*) is successful in real-time ERC, it would imply that we may be able to design specific dynamics in real-time ecological dynamics. Developing a method for efficient ecosystem management is a pressing but challenging task and an ERC-based method might be a basis of a novel approach to manipulate ecological dynamics.

## 3. Conclusion

The present study provides the first empirical evidence that ecological dynamics may possess computational capabilities, and we demonstrate how it can be used in terms of RC. In the context of computational science, ERC does provide a novel framework for computing, and its potential is worth exploring. In the context of ecology, introducing the concept of ‘computational capability’ into ecological studies may open up new research directions. While how and why the computational capability of ecological dynamics evolved cannot be understood in the present study, answering this question may deepen our understanding of how ecological dynamics is driven and how ecosystem functions are maintained, which may contribute to better management and forecasting of ecological dynamics.

## 4. Methods

This section provides the summary of methods, and full methods are described in the electronic supplementary material. Analysis codes and raw data are available at Github (https://github.com/ong8181/ecological-reservoir-computing) and archived at Zenodo (https://doi.org/10.5281/zenodo.7760773 [33]).

### 4.1. A classic reservoir computing framework: echo state network

In the early 2000s, echo state networks (ESNs) as well as liquid state machines (LSMs) were proposed as a seminal reservoir computing (RC) approach [7,34]. ESNs (and LSMs) are different from conventional recurrent neural networks (RNNs) in that weights on the recurrent connections in the reservoir are not trained, but only the weights in the readout are trained [7]. To apply a simple machine learning method to the readout, the reservoir should be appropriately designed in advance. The characteristics of ESNs are briefly described in equation (2.1). Equation (2.1) represents a non-autonomous dynamical system forced by the external input ***u****_t_*. The output is often given by a linear combination of the neuronal states as follows: ***z****_t_* = ***W***_out_***X****_t_*, where ***z****_t_* is the output vector and ***W***_out_ is the weight matrix in the readout. In supervised learning, this weight matrix is trained to minimize the difference between the network output and the desired output for a certain time period.

### 4.2. Demonstration of the concept of ecological reservoir computing

We demonstrate the concept of ERC using a toy model that is frequently used in ecology. Equation (4.1) shows two coupled difference equations that can be interpreted as a model of two-species population dynamics

4.1 $$\begin{array}{rl} & x_{t+1}=x_{t}(r_{x}-r_{x}x_{t}+\beta_{xy}y_{t})\\ & \begin{array}{c} y_{t+1}=y_{t}(r_{y}-r_{y}y_{t}+\beta_{yx}x_{t}), \end{array} \end{array}$$

where *x_t_* and *y_t_* indicate a population density of species *x* and *y* at discrete time *t*, respectively. *r_i_* indicates the population growth rate of species *i*, *β_ij_* indicates influences from species *j* to species *i* (i.e. interspecific interactions), and the second term in the parenthesis indicates density-dependent effects. We used the same *r_i_* for the linear term and squared term following the previous study [19].

The simple nonlinear model can be used as a small reservoir (i.e. reservoir size = 2) in the context of RC. First, any inputs, ***u****_t_*, can be converted using the weight matrix for the input-reservoir connections, ***W***_in_, and then reservoir dynamics follow equation (4.2). This information processing can be described as follows:

4.2 $$\begin{array}{c} X_{t+1}=f(W_{\mathrm{in}}u_{t}+g(X_{t})), \end{array}$$

where *t* denotes discrete time, ***X****_t_* (= {*x_t_*, *y_t_*}) is the state vector of the reservoir and *g* is the two-species population dynamics model (equation (4.1)). *f* represents an element-wise activation function. While hyperbolic tangent is often used as *f* for ESN, here we choose an identity function as *f* so that equation (4.2) can be interpreted as the population dynamics of two species in response to the addition or removal of individuals of species *x* and *y* (thus, equation (4.2) is not ESN, but used here to explain the population dynamics may be regarded as an analogue of ESN). Then, the reservoir states, ***X_t_***, are used to train readout weights by a ridge regression. We used the following parameter values that were determined by a grid search of *r_x_*, *r_y_*, *β_xy_* and *β_yx_*: *r_x_* = 3.0, *r_y_* = 2.7, *β_xy_* = –0.2, *β_xy_* = 0.2. The sparsity (i.e. the proportion of 0 in the matrix elements) of ***W***_in_ (= a matrix that transforms the input value) was set to 0. Matrix elements of the input weight were chosen from a uniform random distribution [–1,1], and they were multiplied by 0.3 to adjust the influence of the input vectors. Readout was trained using a ridge regression (*λ* for the regularization = 0.05). For detailed information of the parameters, see electronic supplementary material, tables S1 and S2. Also, see electronic supplementary material, figure S1a for an example of the dynamics.

This framework enables transforming a traditional ecological population model into an RC system. We used Lorenz attractor as an input, ***u****_t_*, and this simple reservoir predicts one-time-step future of the chaotic time series better than a simple ridge regression (electronic supplementary material, figure S1b–e).

### 4.3. *In silico* ERC: empirical ecological time series and scenario exploration

In *in silico* ERC, an empirical ecological time series is used to simulate an ecological system's responses. For this purpose, we developed a framework based on SSR, a method to reconstruct an original dynamics from a single time series [15,22] (figure 2*a*). In Deyle *et al*. [21], a numerical method to predict an ecosystem's responses to external forces called ‘scenario exploration’ was proposed based on SSR (figure 2*a*). We used the scenario exploration to simulate an ecosystem's response to hypothetical inputs and used the simulated responses as reservoir states. In other words, rules/mechanisms governing the reconstructed ecosystem dynamics were used as a computational resource. The scenario exploration-based *in silico* ERC is fully explained in electronic supplementary material, methods and visually explained in figure 2*a* and electronic supplementary material, appendix I. The *in silico* ecological reservoir shows ESP and possesses a specific memory capacity (figure 3 and electronic supplementary material, methods).

The performance of *in silico* ERC was evaluated using three tasks: prediction of chaotic dynamics, emulation of nonlinear autoregression moving average (named NARMA2, 3, 4, 5 and 10; eqns. S3–S5 in electronic supplementary material were used to generate NARMA2, 3–5 and 10, respectively) time series [35], and generation of an autonomous system (Mackey–Glass equation). All parameters used in the tasks are described in electronic supplementary material, methods and tables S1 and S2.

The performance of *in silico* ERC was compared with that of ESN. ESN was implemented following equation (2.1) and Jaeger [7,16], and detailed parameters are described in electronic supplementary material, tables S1 and S2. The reservoir size (*N* = 2000) was chosen because it showed the highest performance based on our preliminary analysis that tested the effects of reservoir size on the performance (i.e. reservoir sizes from 20 to 2000 were tested). The implementation of *in silico* ERC can be found in the ‘01_ERCinsilico’ folder at the Github repository.

### 4.4. Real-time ERC: a target unicellular microbe

In real-time ERC, real-time ecological dynamics is used as a reservoir. In the present study, the population dynamics of *Tetrahymena thermophila* was used as a reservoir. *Tetrahymena thermophila* (hereafter, *Tetrahymena*) is a unicellular, eukaryotic organism that belongs to the ciliates [26]. *Tetrahymena* is commonly found in a freshwater ecosystem, and is widely used as a model organism in molecular biology studies. *Tetrahymena* can easily be cultured using a wide variety of media, chambers and conditions, and its doubling time is *ca* 2 h under optimal conditions [26]. More detailed physiological characteristics are shown in electronic supplementary material, figures S3 and S4. Experimental conditions to incubate and maintain *Tetrahymena* are described in the subsection ‘A target unicellular organism for real-time ERC: *T. thermophila*’ in electronic supplementary material, methods.

### 4.5. Real-time ERC: *Tetrahymena* population dynamics as a reservoir

Although there is only a single species in the system, the population dynamics at the bottom of the aluminium chamber is a result of complex interactions among biotic and abiotic factors such as medium temperature, cell physiological states, cell–cell interactions and behaviours. Indeed, previous studies demonstrated that *Tetrahymena* population dynamics and behaviour may be influenced by temperature and medium concentrations, and complex nonlinear interactions seem to govern the dynamics and behaviour [27–29]. These studies imply that complex, but deterministic, nonlinear interactions drive the population dynamics of *Tetrahymena*. Specifically, the cell dynamics can be formulated as

4.3 $$\begin{array}{c} X_{t+1}=f_{\mathrm{tetra}}(W_{\mathrm{in}}u_{t},X_{t}), \end{array}$$

where ***W***_in_ determines how the effects of temperature (a scalar value), *u_t_*, propagate to the population dynamics and *f*_tetra_ determines how temperature influence (***W***_in_*u_t_*) and population density captured at the bottom of the chamber (***X****_t_*) interact in the chamber. Importantly, we do not know exact formulations of *f*_tetra_ and ***W***_in_, but we can still use this system for RC if *f*_tetra_ and ***W***_in_ are time-invariant.

### 4.6. Real-time ERC: experimental system to monitor *Tetrahymena* population dynamics

The computational capability of RC positively correlates with the reservoir size, and to increase the reservoir size, we used three concentrations of modified Neff medium (see electronic supplementary material, methods) in the experiments (‘low nutrient,’ ‘med. nutrient’ and ‘high nutrient’; see electronic supplementary material, methods). The *Tetrahymena* population in the medium was incubated in an aluminium chamber (figure 5*a–d*), and the temperature inside the aluminium chamber was automatically regulated using a custom temperature regulator system (E5CC; OMRON, Kyoto, Japan). A user can set a maximum of 256 consecutive temperature values at flexible time intervals. Medium temperatures were changed every 5 min because it took some time to change the medium temperature, and thus the total incubation time for each experiment was 256 time steps × 5 min = 1280 min. The medium temperature was also monitored every minute using a temperature logger/sensor (Ondotori TR-52i; T&D, Matsumoto, Japan). During the incubation, images of the *Tetrahymena* population at the bottom of the aluminium chamber were taken every minute, resulting in 1280 images for each run. Experimental conditions during the monitoring are described in the subsection ‘A target unicellular organism for real-time ERC: *T. thermophila*’ in electronic supplementary material, methods.

### 4.7. Real-time ERC: monitoring *Tetrahymena* population dynamics, preprocessing the cell count data and reservoir state multiplexing

In the experiment, we used four time series as inputs, *u_t_*: (i) uniform random, (ii) Lorenz attractor, (iii) empirical fish-catch time series (flatfish; *Paralichthys olivaceus*), and (iv) empirical fish-catch time series (Japanese jack mackerel; *Trachurus japonicus*). The first one was used to quantify the memory capacity of the ecological reservoir (figure 7), and the other three were used to test the predictive capability of the *Tetrahymena* reservoir (figure 8).

Responses of the *Tetrahymena* population to changing medium temperatures were monitored semi-automatically using a time-lapse camera (figure 5) and the number of *Tetrahymena* cells was counted using a standard particle analysis (electronic supplementary material, figure S5). We preprocessed the raw data so that the time series is stationary and unbiased (electronic supplementary material, figure S5). Then, time- and space-multiplexing techniques were applied to increase the reservoir size (see electronic supplementary material, appendices II and III for visualized explanation of the time- and space-multiplexing). For explanation purposes, we name the reservoir state $S_{t}^{i,j}$, where *i*, *j* and *t* indicate a nutrient condition (‘*l*’, ‘*m*’ and ‘*h*’ denote low, medium and high, respectively), a replicate of the experiment (1 or 2), and time step, respectively. For example, $S_{t}^{m,1}$ indicates the reservoir state taken from the first run of the medium nutrient concentration (4% modified Neff). For the quantification of memory capacity (uniform random value inputs), $\left\{S_{t}^{l,1},S_{t}^{m,1},S_{t}^{h,1}\right\}$ was used for the training and $\left\{S_{t}^{l,2},S_{t}^{m,2},S_{t}^{h,2}\right\}$ was used for the testing. As each *Tetrahymena* time series, $S_{t}^{i,j}$, was time- and space-multiplexed for each run, the combined reservoir state, $\left\{S_{t}^{l,1},S_{t}^{m,1},S_{t}^{h,1}\right\}$, has a 256-row × 15-column matrix (i.e. the experiment generated data that include 256 steps × 5 min (1 image per 1 min) × 3 runs). ***W***_out_, a 1-row × 15-column matrix, was learned by a ridge regression and used to predict a past input value with the test reservoir state, $\left\{S_{t}^{l,2},S_{t}^{m,2},S_{t}^{h,2}\right\}$ (see electronic supplementary material, appendix II for visual explanation). For the prediction tasks, all six reservoir states were time- and space-multiplexed and combined. Thus, $\left\{S_{t}^{l,1},S_{t}^{l,2},S_{t}^{m,1},S_{t}^{m,2},S_{t}^{h,1},S_{t}^{h,2}\right\}$ is a 256-row × 30-column matrix. ***W***_out_ was learned by a ridge regression, and the remaining data were used for testing (see electronic supplementary material, appendix III for visual explanation). Detailed information on the size of training and testing data and training parameters (e.g. ridge regression parameters) is described in electronic supplementary material, methods and table S1. Analysis codes for real-time ERC are available in the ‘02_ERCrealtime’ folder of the Github repository.

## Data accessibility

Analysis codes and data are available at Zenodo (https://doi.org/10.5281/zenodo.7760773 [33]). Preprint: This manuscript was posted as preprint (https://doi.org/10.1101/2021.09.15.460556 [36]).

The supporting data are also provided in electronic supplementary material [37].
